# Subjective Assessment of Objective Image Quality Metrics Range Guaranteeing Visually Lossless Compression

**DOI:** 10.3390/s23031297

**Published:** 2023-01-23

**Authors:** Afnan, Faiz Ullah, Yaseen, Jinhee Lee, Sonain Jamil, Oh-Jin Kwon

**Affiliations:** Department of Electronics Engineering, Sejong University, Seoul 05006, Republic of Korea

**Keywords:** image compression, visually lossless, JPEG 1, image quality assessment, subjective and objective evaluation

## Abstract

The usage of media such as images and videos has been extensively increased in recent years. It has become impractical to store images and videos acquired by camera sensors in their raw form due to their huge storage size. Generally, image data is compressed with a compression algorithm and then stored or transmitted to another platform. Thus, image compression helps to reduce the storage size and transmission cost of the images and videos. However, image compression might cause visual artifacts, depending on the compression level. In this regard, performance evaluation of the compression algorithms is an essential task needed to reconstruct images with visually or near-visually lossless quality in case of lossy compression. The performance of the compression algorithms is assessed by both subjective and objective image quality assessment (IQA) methodologies. In this paper, subjective and objective IQA methods are integrated to evaluate the range of the image quality metrics (IQMs) values that guarantee the visually or near-visually lossless compression performed by the JPEG 1 standard (ISO/IEC 10918). A novel “Flicker Test Software” is developed for conducting the proposed subjective and objective evaluation study. In the flicker test, the selected test images are subjectively analyzed by subjects at different compression levels. The IQMs are calculated at the previous compression level, when the images were visually lossless for each subject. The results analysis shows that the objective IQMs with more closely packed values having the least standard deviation that guaranteed the visually lossless compression of the images with JPEG 1 are the feature similarity index measure (FSIM), the multiscale structural similarity index measure (MS-SSIM), and the information content weighted SSIM (IW-SSIM), with average values of 0.9997, 0.9970, and 0.9970 respectively.

## 1. Introduction

Nowadays, it is a common practice to collect and share a great number of pictures due to advancements in image-acquiring devices such as digital cameras, smartphones with high-definition image-capturing capabilities, and social media platforms [1]. Therefore, there is always a need for efficient image compression techniques to compress this huge amount of image data to reduce its storage size and reduce transmission costs [2]. On a daily basis, vision sensors capture billions of images, which are compressed with an image codec before they are stored or transferred. In fact, image compression plays the role of a fundamental tool which makes it possible to store and share an extensive amount of digital data, such as images and videos [3]. No doubt, image compression is a useful tool, however, while reconstructing images, lossy compression standards may cause some distortions in images that the human eye can detect while comparing the reconstructed images to the originals [4]. The intensity of this alteration in image quality depends on the type of media, the compression level to which the image has been compressed, and other display and environment perspectives [5]. Image compression techniques cause different types of visual abnormalities in images, such as blocking artifacts, color shift, blurring effects, and ringing artifacts, that result in the degradation of the image quality [6]. Therefore, while introducing a new image compression technique, quality assessment techniques should be used to evaluate the performance and to consider the severity of the visual abnormalities produced [7].

To assess the visual quality of the compressed images generated, both objective and subjective methods of image quality evaluation are used [8]. These two types of methods are mentioned in many studies, and are used both in traditional and learning-based image codecs performance evaluation [9]. In an objective case, the image quality is assessed by calculating IQMs that quantitatively assess the image quality. The objective metrics are mathematical models that calculate the image quality precisely and instinctively. The performance of objective metrics is considered a standard that represents a quality performance that is the same as that of human subjects. Several IQMs are defined based on the availability of reference images [10]. In a subjective case, a group of subjects observe image quality subjectively and present their opinion based on the observed image quality [11]. To conduct a standardized subjective test of images, several recommendations are proposed that, when followed, deliver outstanding results [12]. Objective methodologies of image quality evaluation are considered quick and economical, while subjective methods are considered time-consuming and expensive. Further, subjective methods are dependent on the physical conditions and emotions of the viewers, which makes them impractical in real-life applications. However, subjective methods of evaluation are considered more reliable and robust because they mainly rely on the opinions of human subjects, who represent the ultimate users of digital media applications [13].

In the current era, the availability of advanced image-capturing and display devices has increased the interest of researchers to design lossless image compression techniques [13]. Human eyes cannot perceive the very tiny artifacts that appear in compressed images when reconstructed up to a specific compression level. Therefore, trustworthy methodologies for standardization of the evaluation strategies for the visually or near-visually lossless compression standards are released by the joint photographic expert’s group (JPEG) [14]. Aiming to create a solution for visually lossless compression assessment, this paper integrates both of the aforementioned subjective and objective methods of IQA to evaluate the objective metrics, guaranteeing an image’s visually lossless compression with the JPEG 1 standard. For the subjective case, the two alternative forced choice (2AFC)-based strategy is adopted, in which the subject has to determine the visual difference between two images. Human subjects analyze the two test images subjectively through a unique 2AFC-based flicker test method at different compression levels. The compression level is degraded by the subject up to a just noticeable difference level when the subject observes the flickering or visual difference between the original and the corresponding compressed image. The IQMs are determined for the relative images at the point of the previous compression level, at which the images were visually lossless for the particular subject during the flicker test. To perform this subjective flicker test and to calculate the objective metrics, a novel platform “Flicker Test Software” was developed that effectively compressed the images using the JPEG 1 standard at different compression levels to perform the flicker test, then calculated objective IQMs. Furthermore, the results of the objective IQMs that best define the visually lossless compression of the images are discussed. The contributions of this work are summarized in the following points.

1. This study performs a subjective quality assessment of JPEG 1 standard compressed images and evaluates the objective IQM values range that guarantees the visually or near-visually lossless compression of the images.
2. A unique platform “Flicker Test Software” is designed that compress the images using the worldwide utilized JPEG 1 standard at different compression levels to perform a flicker test for the subjective assessment of visually or near-visually lossless compressed images and evaluates the objective IQMs.
3. A subjective test activity performing the flicker test is conducted by 25 participants, individually assessing ten raw images subjectively at different quality levels of compression. The objective metrics for the test images at the point of visually or near-visually lossless compression level observed by each subject are determined.
4. The objective IQMs, named FSIM, MS-SSIM, and IW-SSIM, show the least standard deviations with a close range of values that best guarantee the visually or near-visually lossless compression of the images.

The rest of the paper is organized as follows: Section 2 details the related works and discusses previous IQA methods. Section 3 describes the implementation of the proposed method. In Section 4, the experiments performed in this study are explained and the results are discussed in detail. Section 5 concludes this study and presents future directions.

## 2. Related Work

In the literature, several objective and subjective IQA approaches have been proposed. These IQA methods are used for the evaluation of the high compression to visually lossless image compression models [6]. In the following subsections, an overview of the IQA methods is drawn from the literature.

### 2.1. Objective Image Quality Assessment

The mathematical models are designed to estimate the quality of the image automatically in qualitative terms as observed by human subjects [15]. These metrics are applicable in real-time applications as compared to expensive and time-consuming subjective tests [10]. These metrics have a variety of applications in the field of image processing and computer vision. They can be used in image quality control systems where image quality can be selected based on these quality metrics [16]. Image processing algorithms can be ranked by deploying these metrics to select the algorithm with an output of the highest quality images. In an image communication system of a visual sensors network, these IQMs are used to optimize the filtering procedures at the encoder and decoder end [17].

Studies presented several intelligent image quality measuring metrics that are being utilized in different evaluation studies [18]. These metrics are classified into different categories based on the availability of the absolute quality and distortion-free original image required as an attribute for quality measurement. These are the no-reference, reduced-reference, and full-reference IQMs [19].

#### 2.1.1. No-Reference Image Quality Metrics

For these metrics, the original reference image is not required to calculate the quality of the image [20]. These are the blind IQA metrics that evaluate the visual quality without any reference image. In the blind IQA, the authenticity of the real source distribution and quality distinctions are addressed [21]. The no-reference IQMs calculate image attributes such as luminance, contrast, and other coefficients to predict the image quality. In different image communications platforms, the image quality is evaluated only on the base of the test image without passing its original image as a reference [22]. In comparison with other metrics, prediction of the image quality is complex. The evaluation process of these metrics is also a challenging task, as the original images are unaccounted for in the assessment. These no-reference metrics are integrated into different image evaluation tasks that are briefly discussed in survey papers [23,24,25].

Several no-reference IQA frameworks are proposed based on both the traditional and deep learning methods [9,26]. In this regard, Golestaneh et al. [27] presented a transformer and convolutional neural network (CNN)-based assembled method to rank the images based on local and non-local features. Similarly, Huang et al. [28] proposed a multi-region adjacent pixels correlation (MR-PC) approach to assess the quality of panorama images. Support vector regression (SVR) is used to calculate the difference between the adjacent pixels of the image and to predict the quality measurements. The study of Lee and Park [29] examined the blocking artifacts in images that emerged due to the high-frequency components present on the boundaries of the image and designed a metric verified on the coded images by JPEG.

Furthermore, Mittal et al. [30] proposed a model utilizing the regularized luminance of the images. Similarly, in another study, Mittal et al. [31] proposed a metric named the natural images quality evaluator (NIQE), which incorporated the measurements of the natural scene attributes of an image. Su et al. [32] extracted the image semantics and then adopted the perception rule learning model to predict the image quality. Zhu et al. [33] presented a deep meta-learning-based model trained on generated distortions, which performed efficiently to evaluate the image quality. Ma et al. [34] used the structural and quality features and incorporated a Gaussian mixture model (GMM) and Fisher Vectors (FV). The quality scores were calculated by mapping image descriptions using SVR. Instead of using mean absolute error (MAE) and mean squared error (MSE) loss functions, Li et al. [35] used normalized loss functions based on the Pearson correlation and the root means square error (RMSE). Ying et al. [36] prepared a dataset of distorted images, patches, and subjective qualities, and applied a deep learning-based model in order to predict image quality scores. An adaptive blind IQA framework proposed by Liu et al. [37] utilizes a variety of distortion and quality grades to generate pseudo features. Similarly, Zhang et al. [38] presented an accurate and stable continual learning-based approach trained on different IQA databases. Sun et al. [39] analyzed the relationship between different distortion levels and their types. They presented a distortion graph representation-based deep learning blind IQA approach named “GraphIQA.”

#### 2.1.2. Reduced-Reference Image Quality Metrics

Algorithms for assessing the quality of distorted images that only utilize a limited set of the reference image’s features rather than the entire image are known as reduced reference image quality evaluation metrics [25]. These features are used by the metric as supporting information for predicting the quality of the test image. These attributes possess representation of the reference images and perceptual significance, which are used to evaluate image quality. In this domain, Balanov et al. [40] proposed a discrete cosine transform (DCT) subbands similarity (DSS) quality metric. They performed a spatial downs sampling approach to select the feature for the reference image and maintain good results. Similarly, a structural degradation model (SDM) was proposed that is computationally inexpensive and possesses fast performance [41]. This method acquires the structural degradation information for the original and distorted images and forecasts an image quality score. Further, a reduced referenced image quality metric for contrast (RIQMC) was presented that performs image quality prediction based on the contrast properties of the image [42]. Wu et al. [43] proposed an orientation selectivity-based visual pattern (OSVP) motivated by the human optic system. For metric value, the difference in histograms following different spatial correlated patterns was calculated for the original and the reference images.

#### 2.1.3. Full-Reference Image Quality Metrics

In the case of full reference metrics, the quality of the targeted reconstructed image is assessed by comparing it with its original undistorted image. The value for the full reference metric is calculated by measuring the distortion between the reference and compressed image passed to the metric. Several objective quality metrics following the fully referenced methodology were proposed and discussed in studies [44,45,46]. Using error-based techniques, the mean square error (MSE) and peak signal-to-noise ratio (PSNR) are the highlighted metrics most widely used in evaluation tasks [47,48]. It is not possible to correlate these two metrics with human-based perceptions of image quality, however, which are considered standards in image evaluation tasks [49,50]. In the future, several studies will upgrade the PSNR and overcome its limitations for effective image quality judgment. A metric, weighted signal-to-noise ratio (WSNR), was proposed, which measures the quality by using the contrast sensitivity and weighs the components of the image to predict the human visual system (HVS) [51]. Similarly, an information weighted PSNR (IW-PSNR) was proposed by Wang et al. [52], which utilizes the theoretical principles of information, applying additional weights for graphic content in an image according to their significance.

Further IQMs based on full-reference strategy are the most apparent distortion measure (MAD) [53], the perceptual loss and style score proposed by Johnson et al. [54], the learned perceptual image parch similarity (LPIPS) [55], the perceptual image error assessment via pairwise preference method (PIE-APP) [56], space warping difference network (SWDN) [57]. In terms of structure similarity-based models, the image quality is perceived from the structure information of the images. These metrics include the structural similarity index measure (SSIM) [48], the MS-SSIM [58], the IW-SSIM [52], the edge-based SSIM (E-SSIM) [59], the gradient similarity measure (GSM) [60] and gradient magnitude similarity deviation (GM-SD) [61], the multiscale GMSD (MS-GMSD) [62], the FSIM [63], the visual saliency induced quality index (VSI) [64], the Haar perceptual similarity index (HaarPSI) [65], the mean deviation similarity index (MDSI) [66], the deep image structure and structure similarity method (DISTS) [67], and the visual information fidelity (VIF) metric [68].

### 2.2. Subjective Image Quality Assessment

Subjective methods are considered the most reliable methods for assessing image quality. In these methods, human subjects observe the image quality on displays and signify their opinion about the image according to different types of scales [69]. Subjective tests are performed under the consideration of several standards that are proposed for the trustworthy subjective evaluation of image quality [12,70,71,72]. The international telecommunication union radiocommunication (ITU-R) sector BT.500-11 defined standards for the subjective experiments of television images [12]. These conditions include the testing environment, the displaying methods, and the evaluation of the outcomes of the tests. The ITU-telecommunication (ITU-T) P.910 defined the testing conditions for the subjective analysis of the video data quality [70]. Similarly, ITU-R BT.814-1 defined the contrast and brightness setting of the display devices in the subjective test [71]. Further, the ITU-R BT.1129-2 standardized methods for standard video sequences [72]. Overall, the ITU-R presents different standards that can be summarized into two main categories. These are the single stimulus and double stimulus methods. These two methods are categorized based on the stimulus that is used in the subjective test. In the case of a single stimulus method, a single image is presented to the subject for grading, while in a double stimulus method, each subject observes two different images shown side by side. Each of these methods have specific techniques with different grading scales in order to assess the quality of the images.

#### 2.2.1. Single Stimulus-Based Methods

In single stimulus methodologies, images are presented to subjects who perform the test one by one. While observing images, the subject rates the quality of each image before moving on to the next image. This type is considered the simplest because it is conducted very easily, having few steps. In the study of Cheng et al. [73], they used the concept of this single stimulus test and image quality was assessed. Similarly, Sheikh et al. [20] also used this approach in their evaluation study of the full reference-based IQMs. In single stimulus methods, the absolute category rating (ACR) is the type in which the subject is bound to grade the image quality on a scale of five points, which are: bad, poor, fair, good, and excellent. This method requires a long testing time if the number of images is high. The image content in the stimulus also influences the subject’s opinion. An absolute category rating with hidden reference (ACR-HR) is also used, where the original undistorted image is also included in the stimulus presented to the subject without being known to them. This results in variance removal due to the subject’s opinion of the image content, and diffraction mean opinion scores are calculated. In studies [20,59], this ACR-HR approach is used for the evaluation of the learning-based image codecs. Further, the single stimulus continuous quality evaluation (SSC-QE) is also introduced in the single stimulus category, where a continuous scale is used for grading rather than the discrete scale of one to five. Similarly, Ascenso et al. [74] reviewed the learning-based methods of image coding and performed subjective evaluation experiments using these double stimulus methods.

#### 2.2.2. Double Stimulus-Based Methods

In double stimulus methodologies, two different stimuli are displayed for the person while performing a subjective test for the evaluation of distortion in the images. The techniques for image grading are different in these methods, depending on the test methods. In terms of the time required for the test, double stimulus methods of testing require longer times as compared to single stimulus tests. In these tests, the observer is asked to observe two stimuli for each image. However, double stimulus methods are considered much more reliable and efficient for observing distortion in images. These methods were used by Testolina et al. [19] in their work in which they evaluated the coding performance. The double stimulus impairment scale (DSIS) is an important type of double stimulus method in which a subject observes two images side by side and grades the impairment of the tested image with the second reference original image according to a quality scale of: very annoying, annoying, slightly annoying, perceptible but not annoying, and imperceptible. In this method, the reference image is known to the observer and is always fixed at one position. These methods are also used in state-of-the-art subjective evaluation studies of image quality [74]. In a double stimulus continuous quality scale (DSCQS), the subject conducting the test is bound to grade the quality of both images on a continuous scale. In this test, the reference and the test images are presented randomly. These methods are also time-consuming because the subject observes the images and grades them at each step. In the case of the double stimulus comparison scale (DSCS), the test image is compared with the second original image and graded on a scale of: much worse, worse, slightly worse, the same, slightly better, and much better. DSCSs are also time-consuming, but are considered to have the most reliable performance quality for subjective evaluation.

The above-discussed methods are mostly based on the control environment specified for performing subjective tests. This is the most used method for subjective tests, in which the tests are conducted in a room with normal lighting. This environment helps to eliminate the uncertainties that can result from the influence of the outside environment or other lighting effects. However, crowdsource-based methods were also used for subjective tests instead of the controlled environment conditions. In a study, Egger et al. reviewed [75] the crowdsourced-based methodologies used in the past for IQA. This method was adopted by Chen et al. [76] in their evaluation study. Recently, Testolina et al. [19] performed crowdsourcing-based subjective tests for the evaluation of the learning-based methods using the online platform known as Amazon Mechanical Turk. In crowdsourced environments, the subjects conduct the subjective test remotely in whichever type of environment is available to them.

### 2.3. Subjective Assessment of Visually Lossless Compressed Images

The previously discussed subjective methods are mainly suitable for images with visual distortions that can be easily perceived by human eyes. Recently, high-performance image compression methods have become capable of reconstructing lossless compressed images. Further, with the advancements in storage devices and visual sensor networks, the storage and transportation of a huge amount of data is not a big deal [13]. This leads to a demand for effective image compression algorithms that can provide lossless reconstruction of image data. To standardize these high-performance compression methods, the previous subjective methods discussed are not applicable. While using these approaches, it is almost impossible to notice slight distortions or color swifts in images. In this regard, the JPEG committee has launched standardized methodologies for the effective assessment of high-performance reconstructed visually lossless images. In one case, two test images are presented to the subject along with the original image and the user has to select the least similar image to the original image at a particular time. Similarly, in another case, both the original and reconstructed images are presented on a screen in the same position for the user. These images are interleaved at certain intervals of time. In case of noticeable distortion in the test image, the subject observes some flickering. If the distortion among the images is not perceptible, the user will be unable to observe any type of flickering [14]. In a study by Willème et al. [77], the concept of flickering test methodology was used to evaluate the JPEG XS standard. Recently, Lin et al. [11] used the flicker in the crowdsourced-based subjective test to perceive tiny artifacts. A study by [78] compared compressed images and their corresponding original images by using the flicker test method. The subjects observed the flickering between the two pairs of images. Based on the concept of the flicker test, this study presents a unique subjective test for performing the subjective test of visually lossless compressed images.

## 3. Proposed Methodology

This section of the paper provides a complete overview of the proposed method for the subjective and objective IQMs evaluation of visually lossless image compression. For the subjective evaluation, the flicker test procedures proposed by the JPEG committee for visually or near-visually lossless compressed images are incorporated, and a novel 2AFC-based flicker test is presented [14].

In this proposed framework, the novel “Flicker Test Software” is developed using MATLAB (R2022b) and Unity3D (2021.3.3f1) to conduct the subjective test and calculate the objective metrics for the evaluation of the visually lossless compressed images. The subjective test approach is related to the psychophysical-based adaptive staircase method that is incorporated for the barely noticeable difference in the experimental analysis [79]. In this method, the observer starts from a particular threshold and observes the change in the stimulus. The intensity of the threshold is changed each time and the observer makes a decision based on the difference. This process continues until the stimulus becomes too weak, the difference becomes visible to the observer, and the decision is changed.

In this proposed method, the subject compresses the images using JPEG 1 standard with the highest quality factor and subjectively observes the reconstructed and original image, then observes the visual difference using the 2AFC-based flicker test. The subject decreases the quality factor step-by-step up to the level when he or she observes the visual difference between the original and its reconstructed image.

**Figure 1:**
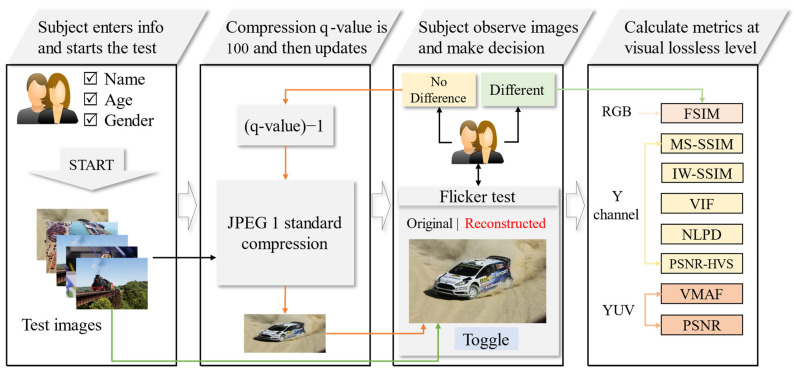
The overall framework of the proposed flicker test for performing subjective assessment and IQMs evaluation at visual lossless compression level. The subject enters his/her information and starts the test. The program selects an image from the test images directory and performs JPEG 1 compression at a quality value (q-value) equal to 100, then displays both the original and reconstructed image in the Unity flicker test, where the subject observes the visual artifacts in the images by toggling the images. When the subject does not observe any flickering, then the image is reconstructed at a lower q-value. In case the subject observes a difference, the IQMs are calculated at the previous q-value (visually lossless stage) and moved to the next image.

For the compression task, one of the most popular and widely used standards, JPEG 1, is employed [80]. In multimedia technologies, JPEG 1 has become one of the most successful compression standards used across the world. JPEG 1 is used for compression tasks in diverse applications such as by digital cameras for photography, in medical images, by web-based applications, for multimedia storage, etc. For performing JPEG 1 compression, the open source “libjpeg-turbo” JPEG image codec is utilized, which can be accessed on the JPEG official website [81]. The overall framework of the proposed method and its workflow is presented in Figure 1. Further description of the “Flicker Test Software” is explained in the following section.

### Flicker Test Software

**Figure 2:**
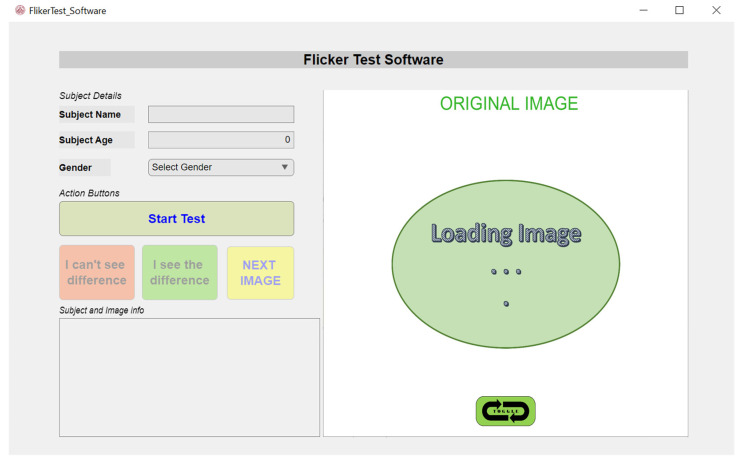
The user interface of the developed “Flicker Test Software”.

The visually or near-visually lossless compressed images have very tiny artifacts that can not be observed by human eyes in normal conditions. To subjectively observe these small changes, the flicker test is a promising solution and has been used by researchers for IQA [14]. The developed “Flicker Test Software” has two parts: first, the selected image is encoded and decoded with the JPEG 1 compression standard using “libjpeg-turbo” implemented in MATLAB, and second, this reconstructed image and the corresponding original image are displayed in a flicker viewer designed in Unity3D for subjective evaluation. Figure 2 shows the interface of the developed framework.

To conduct the subjective test using “Flicker Test Software”, the subject enters his or her details (name, age, and gender) and starts the test. At this step, the current image in the hierarchy is reconstructed with the JPEG 1 standard at the maximum q-value. These reconstructed JPEG 1 images are compressed, and their corresponding original images are displayed at the same coordinates in the designed image viewer using Unity3D. The subject shuffles these images with a toggle button and observes the flickering occurring while shuffling both images in the same position. In case no flickering is noticed, the subject downgrades the quality level and observes the flickering with the newly reconstructed image again. Finally, when the observer notices flickering in the images between the original and reconstructed images at a particular compression level, the objective metrics for the images are calculated at the previous q-value, when the images were visually lossless for the subject conducting the test. Consequently, the subject moves to the next image and conducts the subjective test again for all the test images assigned.

## 4. Experimentation and Results

This section briefly describes the experimental setup of the proposed method, the selected test images, and the evaluation of the IQMs guaranteeing the visually or near-visually lossless compression of images.

### 4.1. Experimental Setup and Display Configuration

The recommendations proposed in ITU-R BT.500-11 in terms of system and display configurations for the subjective assessment standards are followed [12]. These tests are conducted in the controlled environment of the laboratory under controlled lighting conditions. The system is connected to a BENQ monitor, model PD3200U having a size of 32 inches and a resolution of 4K ultra-high-definition. The images are displayed in their actual size to avoid the distortion produced due to the display device. While conducting the test, the subjects are allowed to sit at their preferred comfortable viewing distance according to the display size.

#### 4.1.1. Test Subjects

In case of subjective assessment, twenty-five subjects participated and performed the subjective flicker test. Most likely, the subjects were research students who were used to multimedia applications and had knowledge of image quality and artifacts. However, before starting the test, each student was briefed on the subjective test and the software in order to get used to the procedure, then they performed a demo test. The subjects were guided to perform the test in a relaxed state to obtain authentic results. The subjects were not bound to any time limit; however, the time taken by the subject to perform a single test was determined. At the end of the test, a gift was provided to every participant.

#### 4.1.2. Test Images

**Figure 3:**
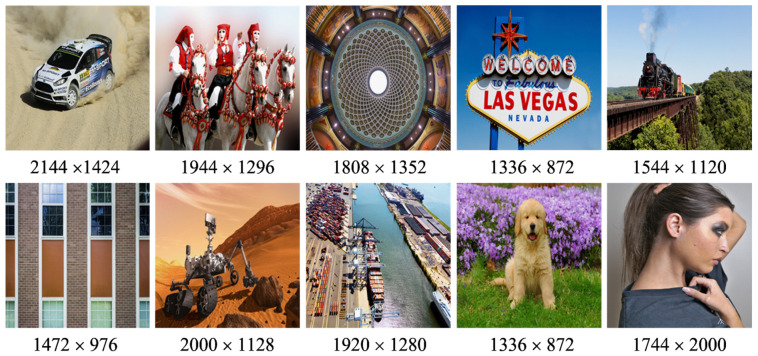
The thumbnails and resolutions of selected raw images from the JPEG-AI test dataset for the subjective test.

In the case of test images, ten raw images were used for the subjective test. These images were selected from the well-known JPEG-AI test dataset that is commonly used for assessment tasks of the image compression frameworks [82]. These images provide a balanced set of different types and categories in terms of image content and spatial resolution. Figure 3 shows the visuals of the selected ten images used for the IQA test.

**Figure 4:**
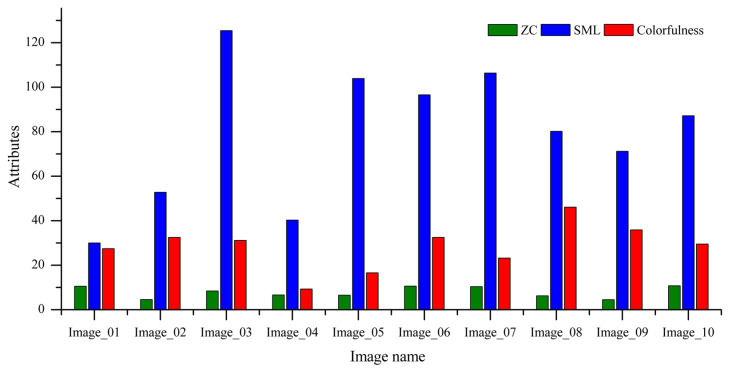
Image quality attributes ZC, SML, and Colorfulness of the test images.

These sample images possess a variety of image quality attributes [83]. The image quality attribute values of the zero crossing (ZC), colorfulness, and sum modified Laplacian (SML) of the selected images are shown in Figure 4, respectively. These graphs show a variety of metric values that guarantee the diversity of the sample images.

#### 4.1.3. Objective Image Quality Metrics

Objective IQMs are calculated for the compressed images at the visually lossless point observed by a particular subject. In this study, we used the well-known IQMs that are used for the assessment of the learning-based image codecs by the JPEG committee during the development of the learning-based image coding standard [84]. Several objective IQMs were evaluated by the JPEG members to find the best-performing metrics in the compression domain based on human perceptions. The suggested IQMs for evaluating compression methods are FSIM, MS-SSIM, IW-SSIM, VIF, the Normalized Laplacian Pyramid (NLPD), PSNR-HSV, VMAF, and PSNR. These IQMs, along with the specified color spaces and channels, are given in Table 1.

### 4.2. Results and Discussion

**Figure 5:**
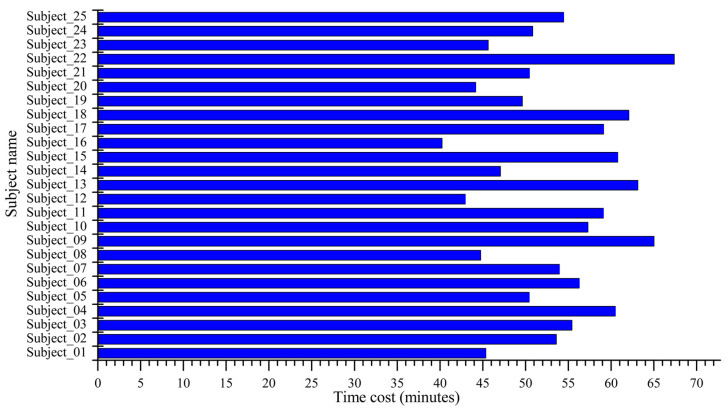
The time cost for each subject conducting the subjective test for the images.

In this section, the resultant data from the subjective and objective assessments are analyzed. In the case of results, the “Flicker Test Software” stored the results for each subject while conducting the flicker test. These data include the information regarding subject and image, test conducting time, and the calculated objective quality metrics for each corresponding image at the visually lossless compression level. Figure 5 shows the time taken by a particular subject to perform the complete single subjective test for the selected images. The average time cost for conducting a single subjective test for the selected images observed in the proposed study is fifty-three minutes.

Table 2 shows the noted q-value and bits per pixel (bpp) recorded as the results of the subjective flicker test. These values are at the point where the images are visually lossless for the subjects while conducting the subjective flicker test. Test images with corresponding minimum q-value (Min q-value), maximum q-value (Max q-value), and the average of the q-value (Avg q-value) recorded while conducting the subjective flicker test by 25 subjects are presented. Similarly, the Min bpp, Max bpp, and Avg bpp are also presented in the table.

In the overall subjective flicker test, the minimum q-value noted for compression is 65 and the maximum value experienced is 100. Because in a few images, the high-frequency color regions are distorted upon the first compression and are easily perceivable by human eyes. In the case of bpp, the overall minimum bpp value across the flicker test noted is 0.3525 and the maximum bpp is 8.3588. The average bpp value is 1.9502 for the visually lossless compressed images across the flicker test observed by the subjects. These results confirm that the range of the q-value and the bpp are not suitable pillars for guaranteeing the visually lossy compression level of images.

The objective quality metrics calculated for the visually lossless compressed images are presented in Table 3. These metrics are calculated in the prescribed channel and color spaces as mentioned in Table 1. The table presents the corresponding average values of the FSIM, MS-SSIM, IW-SSIM, VIF, NLPD, PSNR-HVS, VMAF, and PSNR by each subject recorded from the flicker test.

The varied nature of the selected images (presented in Figure 4) helps us to present a diverse variety of results. The IQMs presents a diverse range of values to the corresponding images at the visually lossless compression level. The overall average value of the FSIM metric noted is 0.9997, guaranteeing visually lossless compression of the images in the subjective test conducted. Similarly, the overall average MS-SSIM value is 0.9970, the average value noted for IW-SSIM is the same (0.9970), the average value for the VIF metric is 0.9930, and the average NLPD value is 0.0542. The PSNR-HVS and PSNR show average values of 44.65 and 42.08, respectively. The average VMAF value guaranteeing the visual losslessness of the compressed images is 94.83 in the overall flicker test.

**Figure 6:**
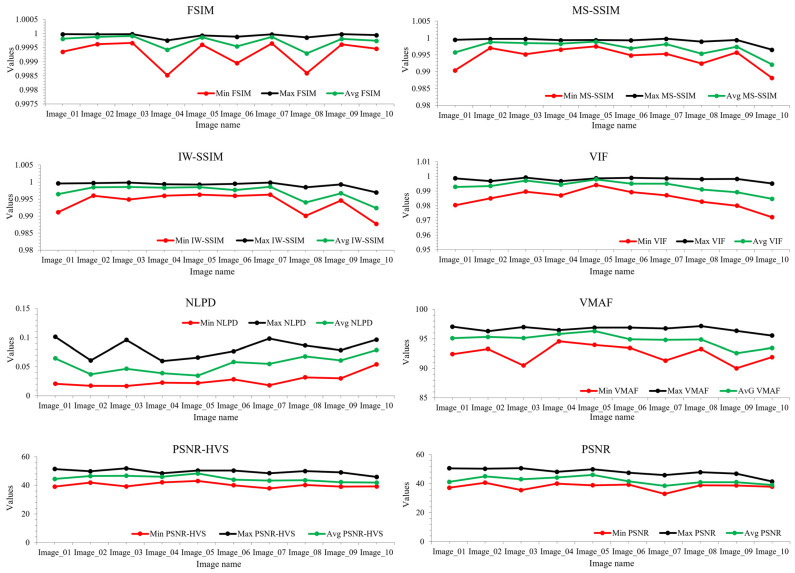
Representation of the line trends of the IQMs for corresponding test images at the point of visually lossless compressed level observed by subjects.

The objective metrics show different trends for the corresponding images. Figure 6 shows the line trends of the objective IQMs for the corresponding images at the stage that are observed as visually lossless by the subjects during the subjective flicker test.

The overall statistical analysis of the IQM values for the test images is presented in Table 4. It shows the overall minimum (Min value), maximum (Max value), average (Avg value), and standard deviation (Std) for the targeted metrics calculated.

The statistical analysis of the objective metrics reveals that the FSIM metric shows the range of the values between the minimum value of 0.9985 to the maximum value of 1.0000, which guarantees the visually lossless compression of the images. The average FSIM value as the outcome of the overall subjective flicker test is 0.9997. As a result, the best metric to guarantee the visual losslessness of the JPEG 1 compressed images is FSIM, with the metrics values at the smallest standard deviation of 0.0003. Next, the best metric that predicts the visual losslessness of JPEG 1 compressed images is MS-SSIM, with an overall average result of 0.9970. It shows a range of values between 0.9882 to the maximum value of 0.9998. These values are almost in the same range, with a standard deviation of 0.0025. In the case of IW-SSIM, it shows a standard deviation of 0.0026, which is the next best metric that guarantees the visual losslessness of the compressed images. The IW-SSIM values are in the range of 0.9877 to 0.9998, with an average of 0.9970 for the particular set of the test images that guarantee visually lossless compression. Further, the VIF also shows satisfying results, with a standard deviation of 0.0054. The VIF shows an overall average of 0.9930. The range of the VIF result values is 0.97722 minimum to 0.09992 maximum. The VMAF values are in the range of 90.01 minimum up to 97.16 maximum. The average of the VMAF is 94.83, with a standard deviation of 1.5799. The performance of the VMAF can also satisfactorily guarantee the visually lossless compression of the images. The performance of NLPD shows an average of 0.0542, with a range of 0.0169 minimum to 0.1014 maximum, and a standard deviation of 0.0209. The performance of the NLPD is not good, with a high range of results as compared to the results of previous metrics. The results of PSNR-HVS fall in the range of 37.8483 minimum and 51.8247 maximum, with an average of 44.6545 at the standard deviation of 3.2799. The PSNR results are in the range of 32.9527 and 50.6389. The average value notified is 42.0773, with a standard deviation of 3.7473.

## 5. Conclusions and Future Work

This paper conducted subjective and objective image quality evaluations for the visually lossless assessment of JPEG 1 compressed images. For this purpose, a platform was developed that accomplished the compression task of images at different quality levels and performed the calculation of IQMs. In the case of the subjective test, a unique concept of the flicker test was used in order to observe the flickering in compressed and reference original images. The subjective activity was performed by 25 students on the test images from the JPEG-AI test dataset. Each image was subjectively observed by all the subjects at different compression levels. The IQMs of the images were calculated at the compression level when the compressed and original images were visually lossless for the subject in the flicker test. The results analysis discussed the range of the quality metrics that guarantee the visually or near-visually lossless compression of the images. The calculated values of the FSIM, MS-SSIM, and IW-SSIM can be effectively utilized with average values of 0.9997, 0.9970, and 0.9970, respectively, to predict the compression level of the images and reconstruct them at the visually lossless compressed quality.

Furthermore, this work can be extended for the performance evaluation of other state-of-the-art image compression algorithms. Moreover, recent IQMs can also be incorporated into the presented framework for further validation. The proposed subjective test methodology can be performed in a crowdsourced-based environment using additional image databases. Our next idea is to integrate the machine and deep learning approaches to perform prediction of the compression level and quality range for reconstructing visually or near-visually lossless compressed images for unknown raw images.

## Figures and Tables

**Table 1:** Objective IQMs and the specified color space and channel used for metric calculation.

S. No.	Objective IQMs	Color Space
1	Feature similarity index measure (FSIM)	RGB
2	Multiscale structural similarity index measure (MS-SSIM)	Y
3	The information content weighted SSIM (IW-SSIM)	Y
4	Visual information fidelity (VIF)	Y
5	Normalized Laplacian pyramid (NLPD)	Y
6	Peak signal-to-noise ratio human visual system (PSNR-HVS)	Y
7	Video multimethod assessment fusion (VMAF)	YUV
8	Peak signal-to-noise ratio (PSNR)	YUV

**Table 2:** Test images with corresponding q-values and bpp values recorded by the flicker test in the proposed subjective test at the point of visually lossless compression.

Image	Min q-Value	Max q-Value	Avg q-Value	Min bpp	Max bpp	Avg bpp
Image_01	69	99	87	1.14	6.42	2.46
Image_02	79	100	92	0.94	4.29	2.09
Image_03	71	100	91	1.74	6.39	3.37
Image_04	78	100	92	0.35	1.99	0.86
Image_05	82	100	95	0.87	3.43	2.23
Image_06	68	97	83	0.53	1.90	0.86
Image_07	73	100	89	2.16	8.36	3.87
Image_08	66	97	80	0.58	2.40	0.96
Image_09	65	95	78	0.88	2.55	1.32
Image_10	67	93	80	0.85	2.74	1.47
**Overall**	**65**	**100**	**86.61**	**0.3525**	**8.3588**	**1.9502**

**Table 3:** The calculated average IQMs for the corresponding images at the point visually lossless compression level in the overall subjective test.

Image	Avg FSIM	Avg MS-SSIM	Avg IW-SSIM	Avg VIF	Avg NLPD	Avg PSNR-HVS	Avg VMAF	Avg PSNR
Image_01	0.9998	0.9958	0.9964	0.9928	0.0644	44.45	95.10	41.21
Image_02	0.9999	0.9988	0.9985	0.9934	0.0369	46.46	95.31	45.06
Image_03	0.9999	0.9985	0.9985	0.9971	0.0466	46.63	95.12	43.02
Image_04	0.9994	0.9983	0.9983	0.9943	0.0391	45.92	95.82	44.23
Image_05	0.9999	0.9989	0.9985	0.9979	0.0348	48.24	96.30	46.05
Image_06	0.9995	0.9970	0.9977	0.9950	0.0581	43.91	94.90	41.58
Image_07	0.9999	0.9982	0.9986	0.9950	0.0548	43.30	94.82	38.51
Image_08	0.9993	0.9954	0.9940	0.9911	0.0677	43.54	94.88	40.98
Image_09	0.9998	0.9974	0.9967	0.9892	0.0609	42.20	92.57	41.03
Image_10	0.9997	0.9922	0.9923	0.9846	0.0786	41.90	93.45	39.10
**Overall**	**0.9997**	**0.9970**	**0.9970**	**0.9930**	**0.0542**	**44.65**	**94.83**	**42.08**

**Table 4:** Statistical analysis of the IQMs for the corresponding test image guarantees visually lossless compression of the images in the flicker test.

**IQMs**	**Min Value**	**Max Value**	**Avg Value**	**Std**
FSIM	0.9985	1.0000	0.9997	**0.0003**
MS-SSIM	0.9882	0.9998	0.9970	**0.0025**
IW-SSIM	0.9877	0.9998	0.9970	**0.0026**
VIF	0.9722	0.9992	0.9930	0.0054
NLPD	0.0169	0.1014	0.0542	0.0209
PSNR-HVS	37.8483	51.8247	44.6545	3.2799
VMAF	90.0126	97.1580	94.8265	1.5799
PSNR	32.9527	50.6389	42.0773	3.7473

## Data Availability

Not applicable.
